# Purity Assessment of Honey Based on Compound Specific Stable Carbon Isotope Ratios Obtained by LC-IRMS

**DOI:** 10.1093/jaoacint/qsae021

**Published:** 2024-03-15

**Authors:** Franz Ulberth, Eric Aries, Oliver De Rudder, Georgios Kaklamanos, Alain Maquet

**Affiliations:** European Commission, Joint Research Centre (JRC), 2440 Geel, Belgium; European Commission, Joint Research Centre (JRC), 2440 Geel, Belgium; European Commission, Joint Research Centre (JRC), 2440 Geel, Belgium; European Commission, Joint Research Centre (JRC), 2440 Geel, Belgium; European Commission, Joint Research Centre (JRC), 2440 Geel, Belgium

## Abstract

**Background:**

The use of stable carbon isotope ratios (δ^13^C) of sugar fractions of honey is a powerful tool to detect adulteration with sugar syrups. This is accomplished by calculating differences of the δ^13^C values between individual honey saccharides and comparing them to published purity criteria. A liquid chromatography–isotope ratio mass spectrometry (LC-IRMS) method for the determination of δ^13^C values of sugars in honey was previously validated by an interlaboratory comparison, but no further guidance was given how to include the obtained precision figures of the compound-specific δ^13^C values in the purity assessment of honey.

**Objective:**

To use existing data to estimate the standard deviation of the repeatability (s_r_) and reproducibility (s_R_) of differences (Δ δ^13^C) between the δ^13^C values of individual honey saccharides.

**Methods:**

Previously published δ^13^C values were used to calculate differences (Δ δ^13^C values) between δ^13^C fructose—δ^13^C glucose, δ^13^C glucose—δ^13^C disaccharides, etc. in a honey sample; s_r_ and s_R_ of Δ δ^13^C values were calculated according to ISO 5725–2:2019.

**Results:**

The Δ δ^13^C s_r_ and s_R_ values were essentially of the same magnitude as the s_r_ and s_R_ values of δ^13^C values of the sugar fractions. The precision of the Δ δ^13^C values was used to estimate the critical difference for comparing a test result with a reference value according to ISO 5725–6:1994. This varied between 0.26 and 1.10‰.

**Conclusion:**

The estimated critical differences can be used to determine whether a honey test result complies with published Δ δ^13^C purity criteria.

**Highlight:**

The proposed procedure will increase confidence in decisions based on compound-specific δ^13^C values regarding the conformity of honey with published purity criteria.

The economically motivated adulteration of honey compromises its quality and integrity and is of increasing prevalence. Among the most prevalent malpractices is the addition of sugar syrups or feeding bees with syrups during the nectar flow to increase volume and lower production costs for financial gain. Such deceptive practices erode the trust and reputation of the honey value chain, leading to consumers mistrusting the manufacturers’ claims of product authenticity, purity, and quality (1). Recognizing the need to address this critical issue, the scientific community has developed advanced analytical methods to detect honey adulteration (reviewed in 2–4). Among them, liquid chromatography–isotope ratio mass spectrometry (LC-IRMS) has emerged as a powerful tool for the detection of honey adulteration with sugars derived from C3 plants (rice, wheat, sugar beet), and increased the sensitivity for detecting sugars from C4 plants (corn, sugar cane) (5–7). The addition of 1–2% sugar syrups derived from C4 plants, a level where the AOAC *Official Method*^SM^  **998.12** fails (8), can be reliably detected using the LC-IRMS method. However, the detection capability for syrups from C3 plants is poorer and varies between 10 and 20%, depending on the type of sugar syrup.

Recently, the LC-IRMS method was validated through interlaboratory comparison (ILC) (9), and the precision of the stable carbon isotope ratios (δ^13^C) of mono-, di-, and trisaccharides formed the basis for the standardization of the method by the European Committee for Standardization (CEN) (10). The standard provides instructions for the determination of δ^13^C values but does not give guidance how the obtained data can be used for the purity assessment of honey. International marketing standards for honey such as the FAO/WHO Codex Alimentarius Standard 12-1981 (11), the EU Honey Directive (12), or the USP Honey Standard (13) do not describe reference values of compound-specific δ^13^C values that can be employed for purity assessment. The most common approach builds on the strong correlations between the δ^13^C values of individual saccharides, which are altered when exogenous sugars are added. For purity assessment, the differences between the δ^13^C values of sugar pairs (Δ δ^13^C) are calculated, i.e., Δ δ^13^C [fructose—glucose], Δ δ^13^C [fructose—disaccharides], etc., and compared to cutoff values estimated by analysis of authentic honey samples. Elflein and Raezke (6) analyzed 451 authentic honeys and determined cutoff values, termed purity criteria, that are ±1‰ for Δ δ^13^C [fructose—glucose], and ±2.1‰ for all other Δ δ^13^C values of LC separated sugar fractions (Δ δ^13^C max). These purity criteria are widely used by private and official control laboratories; yet no agreement exists how to take into account measurement uncertainty in the purity assessment.

The Joint Research Centre (JRC) of the European Commission carried out two larger studies to detect sugar syrups in honey by using LC-IRMS (14, 15), making use of the published purity criteria (6). To take account of the uncertainty of the measured Δ δ^13^C values, the variances of the δ^13^C values of the sugar fractions obtained from a honey quality control material (=intra-laboratory reproducibility) were combined to give

[1] $$\left(\mathrm{uncertainty}\right)\;u=\sqrt{{\mathrm{variance}}_{\mathrm{sugarA}}+{\mathrm{variance}}_{\mathrm{sugarB}}}$$

If the test results minus the expanded uncertainty (k = 2) exceeded the respective purity criteria, the honey was deemed suspicious of adulteration. Obviously, this approach does not take account of between-laboratory sources of uncertainty. Therefore, this article explores the opportunity to use the data from the multi-laboratory validation of the LC-IRMS method to estimate repeatability and reproducibility of Δ δ^13^C values, which can be used for comparing an obtained measurement result with a reference value as described in Clause 4.2.3 of ISO 5725–6:1994 (16).

## Experimental

The setup of the ILC to validate the LC-IRMS method for the determination of δ^13^C values of honey saccharides is described in (9). Most of the 14 participating laboratories employed columns filled with polymeric styrene-divinylbenzene resins in the Ca^2+^ form and followed the protocol described in (6) for separation; one participant used an anion-exchange (CarboPac) column. Honey samples were selected from the JRC collection of honeys (Supplementary Table S1).

Precision (repeatability and reproducibility) of Δ δ^13^C values was estimated according to ISO 5725–2:2019 procedures (17); calculations were done with the AOAC Interlaboratory Study Workbook—Blind Replicates (18).

## Results and Discussion

The precision data of the LC-IRMS method to determine compound-specific δ^13^C values of honey saccharides have already been reported (9). However, the results of such an analysis are usually used to assess honey purity by calculating all possible differences (Δ δ^13^C values) between fructose, glucose, di-, and trisaccharides and comparing them with the purity criteria proposed in (6). In a conformity assessment, the uncertainty of the test results, in this case the Δ δ^13^C values, has to be taken into account. One way of doing this is to combine mathematically an estimate of the uncertainty of δ^13^C values of the two sugars used to calculate the difference (Equation 1). An alternative approach is to use the uncertainty of the calculated Δ δ^13^C values directly, which aligns more closely with the primary objective of the LC-IRMS analysis using the Elflein and Raezke (6) purity criteria. To this end, the collaborative study data were used to calculate the Δ δ^13^C values per laboratory and honey sample. Standard deviations for repeatability (s_r_) and reproducibility (s_R_) were then calculated according to ISO 5725–2:2019 (17). Once s_r_ and s_R_ are known, they can be used to calculate the critical difference (CD) as given in Clause 4.2.3 of ISO 5725–6:1994, which is

[2] $$CD=\frac{1}{\sqrt{2}}\sqrt{{\left(2.8s_{R}\right)}^{2}-{\left(2.8S_{r}\right)}^{2\;}(\frac{n-1}{n})}$$

If the absolute difference between the mean of the Δ δ^13^C values of *n* LC-IRMS measurements and the reference value (purity criterion) exceeds the CD, this indicates a suspect sample at the 95% probability level. A prerequisite for this approach is that the testing laboratory’s s_r_ values are consistent with the precision estimates from the collaborative study. ISO 21748:2017 provides guidance how to verify this requirement (19).

Table 1 summarizes the s_r_ and s_R_ of Δ δ^13^C values calculated from the data obtained in the ILC; Supplementary Tables S1–S7 contain more detailed information on the outcome of the data evaluation. The precision estimates of the Δ δ^13^C values are essentially of the same magnitude as the δ^13^C values of the sugar fractions estimated in the ILC (9) (Table 2).

**Table 1.** Precision data for the differences between δ^13^C values of honey saccharides (Δ δ^13^C, absolute values) and critical differences for comparing the mean of duplicate test results (n = 2) with a reference value: A (lemon), B (polyfloral), C (honeydew), D (honeydew), E (acacia), F (lavender)

	Honey sample						
	A	B	C	D	E	F	Mean
Δ δ^13^C (‰) [fructose—glucose]							
s_r_	0.07	0.10	0.10	0.15	0.07	0.06	0.09
s_R_	0.25	0.43	0.31	0.25	0.21	0.14	0.27
CD	0.49	0.69	0.60	0.45	0.40	0.26	0.48
Δ δ^13^C (‰) [fructose—disaccharides]							
s_r_	0.15	0.14	0.22	0.18	0.13	0.14	0.16
s_R_	0.31	0.34	0.31	0.31	0.29	0.34	0.32
CD	0.57	0.65	0.53	0.56	0.54	0.64	0.58
Δ δ^13^C (‰) [fructose—trisaccharides]							
s_r_	0.15	0.21	0.16	0.27	0.18	0.39	0.23
s_R_	0.26	0.47	0.28	0.31	0.50	0.55	0.40
CD	0.47	0.88	0.51	0.48	0.96	0.94	0.71
Δ δ^13^C (‰) [glucose—disaccharides]							
s_r_	0.14	0.12	0.26	0.12	0.16	0.14	0.16
s_R_	0.24	0.47	0.30	0.37	0.21	0.31	0.32
CD	0.43	0.92	0.47	0.71	0.35	0.58	0.58
Δ δ^13^C (‰) [glucose—trisaccharides]							
s_r_	0.33	0.08	0.27	0.25	0.18	0.22	0.22
s_R_	0.35	0.56	0.40	0.50	0.50	0.44	0.46
CD	0.52	1.10	0.70	0.93	0.96	0.81	0.84
Δ δ^13^C (‰) [disaccharides—trisaccharides]							
s_r_	0.50	0.15	0.11	0.14	0.24	0.40	0.26
s_R_	0.57	0.40	0.25	0.27	0.52	0.54	0.43
CD	0.89	0.76	0.47	0.50	0.97	0.91	0.75

**Table 2.** Precision data for δ^13^C values of fructose, glucose, disaccharides, and trisaccharides determined by LC-IRMS in honey (9): A (lemon), B (polyfloral), C (honeydew), D (honeydew), E (acacia), F (lavender)

	Honey sample					
	A	B	C	D	E	F
δ^13^C (‰) fructose						
s_r_	0.13	0.09	0.09	0.08	0.08	0.12
s_R_	0.38	0.28	0.19	0.27	0.38	0.37
δ^13^C (‰) glucose						
s_r_	0.10	0.10	0.12	0.13	0.12	0.12
s_R_	0.31	0.43	0.34	0.33	0.29	0.42
δ^13^C (‰) disaccharides						
s_r_	0.18	0.08	0.24	0.15	0.14	0.12
s_R_	0.35	0.34	0.36	0.24	0.36	0.31
δ^13^C (‰) trisaccharides						
s_r_	0.68	0.19	0.23	0.25	0.15	0.38
s_R_	0.68	0.36	0.35	0.50	0.45	0.57

**Figure 1.**
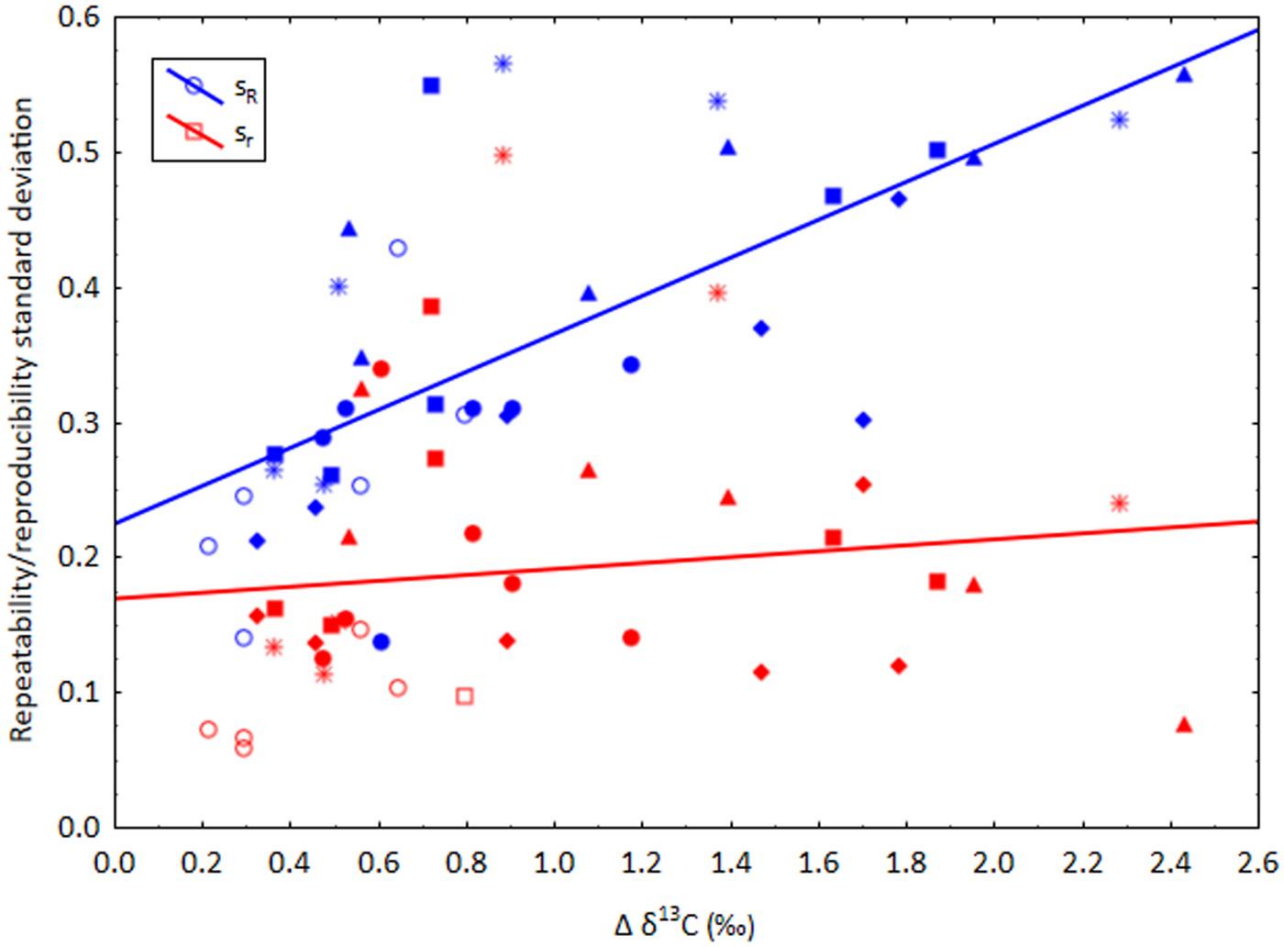
Relationship between the differences (Δ) of the δ^13^C values between the LC separated sugars and the repeatability and reproducibility standard deviation obtained by an interlaboratory comparison (9). Symbols: open circles, Δ δ^13^C [fructose—glucose]; filled circles, Δ δ^13^C [fructose—disaccharides], rectangles, Δ δ^13^C [fructose—trisaccharides]; diamonds, Δ δ^13^C [glucose—disaccharides]; triangles, Δ δ^13^C [glucose—trisaccharides]; stars, Δ δ^13^C [disaccharides—trisaccharides].

The standard deviation of repeatability (s_r_) of Δ δ^13^C values was, with few exceptions, independent of the magnitude of the Δ δ^13^C values (correlation coefficient r = 0.134, *P* > 0.1). On the other hand, the standard deviation of reproducibility (s_R_) increased with increasing Δ δ^13^C values (correlation coefficient r = 0.700, *P* < 0.05). As expected, the Δ δ^13^C values of [fructose—trisaccharides], [glucose—disaccharides], [glucose—trisaccharides], and [disaccharides—trisaccharides] were higher than the Δ δ^13^C values of [fructose—glucose] and [fructose—disaccharides] (Figure 1). The CD values for duplicate analyses (*n *=* *2) calculated according to Equation 2 varied between 0.26 and 1.10‰, with means ranging from 0.48‰ for Δ δ^13^C [fructose—disaccharides] to 0.84‰ for [glucose—disaccharides] (Table 1). Using the mean CD values to compare a test result to the reference value may raise the risk of false-positive assessments. It may, therefore, be more prudent to use the highest CD values for comparison: 0.7‰ for Δ δ^13^C [fructose—glucose] and 1.1‰ for all other differences.

The CD values can also be incorporated into the approach proposed by Elflein and Raezke (6) for assessing the purity of honey. In other words, if the mean of duplicate LC-IRMS analysis of a honey sample results in an absolute Δ δ^13^C [fructose—glucose] value that is greater than 1.7‰ and/or an absolute Δ δ^13^C max value greater than 3.2‰, the sample should be considered noncompliant with the reference values at a confidence level of 95%.

## Conclusions

We propose to include the CD in the evaluation of honey purity based on published Δ δ^13^C purity criteria (6), which takes the uncertainty of δ^13^C values of honey saccharides into account. The CD for Δ δ^13^C [fructose—glucose] was estimated as 0.7‰ and for Δ δ^13^C max as 1.1‰ provided the mean of duplicate LC-IRMS analyses is used for calculating CD. For practical reasons, laboratories may decide to carry out a single determination first, and if δ^13^C [fructose—glucose] and/or Δ δ^13^C max is above the purity criteria, to repeat the analysis for confirming the suspicion.

## Supplementary Material

qsae021_Supplementary_Data
