# Correction to: Purity Assessment of Honey Based on Compound Specific Stable Carbon Isotope Ratios Obtained by LC-IRMS

**DOI:** 10.1093/jaoacint/qsae065

**Published:** 2024-08-28

This is a correction to: Franz Ulberth, Eric Aries, Oliver De Rudder, Georgios Kaklamanos, Alain Maquet, Purity Assessment of Honey Based on Compound Specific Stable Carbon Isotope Ratios Obtained by LC-IRMS, *Journal of AOAC INTERNATIONAL*, 2024; https://doi.org/10.1093/jaoacint/qsae021

Errors were detected after the first publication of the paper (ACCEPTED MS). These are outlined in this notice, as well as completed in the final corrected version of the article.

Incorrect units were inadvertently introduced in sections **Abstract**, Equation [2], **Results and Discussion**, **Conclusion**, and **Table 1**.

In the **Abstract,** under **Results,** the last sentence should read: “This varied between 0.26 ‰ and 1.10 ‰” instead of: “This varied between 0.02 ‰ and 0.40 ‰.”

Equation [2] should be:

$$CD=\frac{1}{\sqrt{2}}\sqrt{{\left(2.8s_{R}\right)}^{2}-{\left(2.8S_{r}\right)}^{2\;}(\frac{n-1}{n})}$$

instead of:

$$CD=\frac{1}{\sqrt{2}}\sqrt{s_{R}^{2}{-s}_{r}^{2}(\frac{n}{n-1})}$$

Under heading **Results and Discussion**, in the penultimate paragraph, the final sentences were changed to read as two:

“The CD values for duplicate analyses (*n*=2) calculated according to equation [2] varied between 0.26 ‰ and 1.10 ‰, with means ranging from 0.48 ‰ for Δ δ^13^C [fructose – disaccharides] to 0.84 ‰ for [glucose – disaccharides] (Table 1). It may, therefore, be more prudent to use the highest CD values for comparison: 0.7 ‰ for Δ δ^13^C [fructose – glucose] and 1.1 ‰ for all other differences.” instead of three:

“The CD values for duplicate analyses (*n*=2) calculated according to equation [2] varied between 0.02 ‰ and 0.40 ‰, with means ranging from 0.14 ‰ for Δ δ13C [fructose – disaccharides] to 0.26 ‰ for [disaccharides – trisaccharides] (Table 1). Using the mean CD values to compare a test result to the reference value may raise the risk of false-positive assessments. It may, therefore, be more prudent to use the highest CD values for comparison: 0.24 ‰ for Δ δ13C [fructose – disaccharides] and 0.40 ‰ for all other differences.”

And under the same heading, the final paragraph was changed to read:

“In other words, if the mean of duplicate LC-IRMS analysis of a honey sample results in an absolute Δ δ^13^C [fructose – glucose] value which is greater than 1.7 ‰ and/or an absolute Δ δ^13^C max value greater than 3.2 ‰, the sample should be considered as non-compliant with the reference values at a confidence level of 95 %.”

instead of

“The CD values can also be incorporated into the approach proposed by Elflein and Raezke (6) for assessing the purity of honey. In other words, if the mean of duplicate LC-IRMS analysis of a honey sample results in an absolute Δ δ13C [fructose – glucose] value which is greater than 1.24 ‰ and/or an absolute Δ δ13C max value greater than 2.50 ‰, the sample should be considered as non-compliant with the reference values at a confidence level of 95 %.”

Under **Conclusions,** the second sentence was changed to read:

“The CD for Δ δ^13^C [fructose – glucose] was estimated as 0.7 ‰ and for Δ δ^13^C max as 1.1 ‰ provided the mean of duplicate LC-IRMS analyses is used for calculating CD.”

instead of:

“The CD for Δ δ13C [fructose – glucose] was estimated as 0.24 ‰ and for Δ δ13C max

as 0.40 ‰ provided the mean of duplicate LC-IRMS analyses is used for calculating CD.”

In **Table 1** all values in all rows labelled CD were changed to read as below:

Instead of:

These changes are included in the final corrected version of the article.

**Table 1.** Precision data for the differences between δ^13^C values of honey saccharides (Δ δ^13^C, absolute values) and critical differences for comparing the mean of duplicate test results (n=2) with a reference value. A (lemon), B (polyfloral), C (honeydew), D (honeydew), E (acacia), F (lavender).

	Honey sample						
	A	B	C	D	E	F	mean
Δ δ^13^C (‰) [fructose-glucose]							
s_r_	0.07	0.10	0.10	0.15	0.07	0.06	0.09
s_R_	0.25	0.43	0.31	0.25	0.21	0.14	0.27
CD	0.49	0.69	0.60	0.45	0.40	0.26	0.48
Δ δ^13^C (‰) [fructose-disaccharides]							
s_r_	0.15	0.14	0.22	0.18	0.13	0.14	0.16
s_R_	0.31	0.34	0.31	0.31	0.29	0.34	0.32
CD	0.57	0.65	0.53	0.56	0.54	0.64	0.58
Δ δ^13^C (‰) [fructose-trisaccharides]							
s_r_	0.15	0.21	0.16	0.27	0.18	0.39	0.23
s_R_	0.26	0.47	0.28	0.31	0.50	0.55	0.40
CD	0.47	0.88	0.51	0.48	0.96	0.94	0.71
Δ δ^13^C (‰) [glucose-disaccharides]							
s_r_	0.14	0.12	0.26	0.12	0.16	0.14	0.16
s_R_	0.24	0.47	0.30	0.37	0.21	0.31	0.32
CD	0.43	0.92	0.47	0.71	0.35	0.58	0.58
Δ δ^13^C (‰) [glucose-trisaccharides]							
s_r_	0.33	0.08	0.27	0.25	0.18	0.22	0.22
s_R_	0.35	0.56	0.40	0.50	0.50	0.44	0.46
CD	0.52	1.10	0.70	0.93	0.96	0.81	0.84
Δ δ^13^C (‰) [disaccharides-trisaccharides]							
s_r_	0.50	0.15	0.11	0.14	0.24	0.40	0.26
s_R_	0.57	0.40	0.25	0.27	0.52	0.54	0.43
CD	0.89	0.76	0.47	0.50	0.97	0.91	0.75

**Table 1:** Precision data for the differences between δ^13^C values of honey saccharides (Δ δ^13^C, absolute values) and critical differences for comparing the mean of duplicate test results (n=2) with a reference value. A (lemon), B (polyfloral), C (honeydew), D (honeydew), E (acacia), F (lavender).

	Honey sample						
	A	B	C	D	E	F	mean
Δ δ^13^C (‰) [fructose-glucose]							
s_r_	0.07	0.10	0.10	0.15	0.07	0.06	0.09
s_R_	0.25	0.43	0.31	0.25	0.21	0.14	0.27
CD	0.16	0.24	0.20	0.09	0.13	0.08	0.15
Δ δ^13^C (‰) [fructose-disaccharides]							
s_r_	0.15	0.14	0.22	0.18	0.13	0.14	0.16
s_R_	0.31	0.34	0.31	0.31	0.29	0.34	0.32
CD	0.16	0.20	0.02	0.12	0.16	0.20	0.14
Δ δ^13^C (‰) [fructose-trisaccharides]							
s_r_	0.15	0.21	0.16	0.27	0.18	0.39	0.23
s_R_	0.26	0.47	0.28	0.31	0.50	0.55	0.40
CD	0.11	0.26	0.12	0.22^*)^	0.30	0.39^*)^	0.23
Δ δ^13^C (‰) [glucose-disaccharides]							
s_r_	0.14	0.12	0.26	0.12	0.16	0.14	0.16
s_R_	0.24	0.47	0.30	0.37	0.21	0.31	0.32
CD	0.10	0.31	0.21^*)^	0.23	0.15^*)^	0.17	0.19
Δ δ^13^C (‰) [fructose-trisaccharides]							
s_r_	0.33	0.08	0.27	0.25	0.18	0.22	0.22
s_R_	0.35	0.56	0.40	0.50	0.50	0.44	0.46
CD	0.25^*)^	0.39	0.08	0.25	0.30	0.22	0.25
Δ δ^13^C (‰) [disaccharides-trisaccharides]							
s_r_	0.50	0.15	0.11	0.14	0.24	0.40	0.26
s_R_	0.57	0.40	0.25	0.27	0.52	0.54	0.43
CD	0.40^*)^	0.24	0.14	0.13	0.28	0.38^*)^	0.26

*) only s_R_ used for estimating CD since $s_{R}^{2}{-s}_{r}^{2}\left(\frac{n}{n-1}\right)\;$gives a negative result

