# Supplementary material for: Purity Assessment of Honey Based on Compound Specific Stable Carbon Isotope Ratios Obtained by LC-IRMS
Source: J AOAC Int. 2024 Mar 15;107(5):884–7. doi: 10.1093/jaoacint/qsae021 (PMC11382942; doi:10.1093/jaoacint/qsae021)
Supplement: qsae021_Supplementary_Data [file qsae021_supplementary_data.docx]

**Supplemental information**

Table S1: Characteristics of honey samples used in the multi-laboratory validation study

| Sample | Characteristic | Indicated by |
| --- | --- | --- |
| A *(*Monofloral - Lemon*)* | Adulterated | Presence of C4 sugars by EA/LC-IRMS |
| B (Polyfloral) | Genuine | Conformity with purity criteria (6) and absence of oligosaccharides |
| C (Honeydew) | Undecided | Presence of oligosaccharides by LC-IRMS but otherwise conforming with purity criteria (6) |
| D (Honeydew) | Adulterated | Presence of oligosaccharides by LC-IRMS and UHPLC-MS |
| E (Monofloral - Acacia) | Undecided | Different δ ^13^C observed in the trisaccharide fraction |
| F (Monofloral -Lavender) | Genuine | Conformity with purity criteria (6) and absence of oligosaccharides |

Table S2: Precision data for the differences (Δ δ^13^C (‰), absolute values) between δ^13^C (‰) of fructose and δ^13^C (‰) of glucose in honey determined by LC–IRMS.

|  | Sample | | | | | |
| --- | --- | --- | --- | --- | --- | --- |
|  | A | B | C | D | E | F |
| Parameter |  |  |  |  |  |  |
| Number of  laboratories | 12 | 12 | 12 | 12 | 12 | 12 |
| Number of laboratories after elimination of  outliers | 12 | 11 | 12 | 12 | 11 | 10 |
| Outliers (Cochran  test) | - | 1 | - | - | 1 | 1 |
| Outliers (Grubbs  test) | - | - | - | - | - | 1 |
| Mean | 0.296 | 0.567 | 0.796 | 0.560 | 0.212 | 0.294 |
| Reproducibility  standard deviation  (sR) | 0.246 | 0.349 | 0.305 | 0.253 | 0.209 | 0.140 |
| Repeatability  standard deviation  (sr) | 0.067 | 0.052 | 0.097 | 0.146 | 0.072 | 0.059 |
| Relative sR (%) | 83.3 | 61.6 | 38.3 | 45.2 | 98.3 | 47.4 |
| Relative sr (%) | 22.7 | 9.2 | 12.2 | 26.2 | 33.8 | 20.1 |
| Reproducibility,  R (2.80 * sR) | 0.691 | 0.98 | 0.853 | 0.708 | 0.58 | 0.391 |
| Repeatability,  r (2.80 * sr) | 0.188 | 0.15 | 0.271 | 0.410 | 0.20 | 0.165 |

Table S3: Precision data for the differences (Δ δ^13^C (‰), absolute values) between δ^13^C (‰) of fructose and δ^13^C ((‰) of disaccharides in honey determined by LC–IRMS.

|  | Sample | | | | | |
| --- | --- | --- | --- | --- | --- | --- |
|  | A | B | C | D | E | F |
| Parameter |  |  |  |  |  |  |
| Number of  laboratories | 12 | 12 | 12 | 12 | 12 | 12 |
| Number of laboratories after elimination of  outliers | 12 | 12 | 12 | 12 | 12 | 12 |
| Outliers (Cochran  test) | - | - | - | - | - | - |
| Outliers (Grubbs  test) | - | - | - | - | - | - |
| Mean | 0.527 | 1.177 | 0.815 | 0.907 | 0.473 | 0.606 |
| Reproducibility  standard deviation  (sR) | 0.310 | 0.343 | 0.310 | 0.310 | 0.288 | 0.339 |
| Repeatability  standard deviation  (sr) | 0.154 | 0.141 | 0.218 | 0.180 | 0.125 | 0.137 |
| Relative sR (%) | 58.8 | 29.1 | 38.0 | 34.1 | 60.8 | 55.9 |
| Relative sr (%) | 29.2 | 12.0 | 26.7 | 19.8 | 26.3 | 22.6 |
| Reproducibility,  R (2.80 * sR) | 0.868 | 0.960 | 0.867 | 0.867 | 0.806 | 0.949 |
| Repeatability,  r (2.80 * sr) | 0.431 | 0.395 | 0.609 | 0.504 | 0.349 | 0.383 |

Table S4: Precision data for the differences (Δ δ^13^C (‰), absolute values) between δ^13^C (‰) of fructose and δ^13^C (‰) of trisaccharides in honey determined by LC–IRMS.

|  | Sample | | | | | |
| --- | --- | --- | --- | --- | --- | --- |
|  | A | B | C | D | E | F |
| Parameter |  |  |  |  |  |  |
| Number of  laboratories | 9 | 12 | 12 | 12 | 9 | 9 |
| Number of laboratories after elimination of  outliers | 8 | 11 | 12 | 11 | 8 | 9 |
| Outliers (Cochran  test) | 1 | - | - | - | 1 | - |
| Outliers (Grubbs  test) | - | 1 | - | 1 | - | - |
| Mean | 0.494 | 1.635 | 0.364 | 0.731 | 1.873 | 0.722 |
| Reproducibility  standard deviation  (sR) | 0.261 | 0.467 | 0.276 | 0.313 | 0.502 | 0.549 |
| Repeatability  standard deviation  (sr) | 0.150 | 0.214 | 0.162 | 0.273 | 0.182 | 0.385 |
| Relative sR (%) | 52.8 | 28.6 | 76.0 | 42.8 | 26.8 | 76.1 |
| Relative sr (%) | 30.4 | 13.1 | 44.4 | 37.3 | 9.7 | 53.3 |
| Reproducibility,  R (2.80 * sR) | 0.729 | 1.307 | 0.452 | 0.763 | 1.404 | 1.537 |
| Repeatability,  r (2.80 * sr) | 0.420 | 0.598 | 0.774 | 0.876 | 0.510 | 1.076 |

Table S5: Precision data for the differences (Δ δ^13^C (‰), absolute values) between δ^13^C (‰) of glucose and δ^13^C (‰) of disaccharides in honey determined by LC–IRMS.

|  | Sample | | | | | |
| --- | --- | --- | --- | --- | --- | --- |
|  | A | B | C | D | E | F |
| Parameter |  |  |  |  |  |  |
| Number of  laboratories | 12 | 12 | 12 | 12 | 12 | 12 |
| Number of laboratories after elimination of  outliers | 12 | 12 | 11 | 12 | 12 | 12 |
| Outliers (Cochran  test) | - | - | - | - | - | - |
| Outliers (Grubbs  test) | - | - | 1 | - | - | - |
| Mean | 0.457 | 1.779 | 1.699 | 1.467 | 0.322 | 0.890 |
| Reproducibility  standard deviation  (sR) | 0.237 | 0.466 | 0.302 | 0.370 | 0.213 | 0.305 |
| Repeatability  standard deviation  (sr) | 0.137 | 0.120 | 0.255 | 0.116 | 0.158 | 0.139 |
| Relative sR (%) | 51.8 | 26.2 | 17.8 | 25.2 | 66.2 | 34.2 |
| Relative sr (%) | 30.1 | 6.7 | 15.0 | 7.9 | 48.9 | 15.7 |
| Reproducibility,  R (2.80 * sR) | 0.662 | 1.304 | 0.846 | 1.037 | 0.597 | 0.853 |
| Repeatability,  r (2.80 * sr) | 0.384 | 0.334 | 0.715 | 0.324 | 0.442 | 0.390 |

Table S6: Precision data for the differences (Δ δ^13^C (‰), absolute values) between δ^13^C (‰) of glucose and δ^13^C (‰) of trisaccharides in honey determined by LC–IRMS.

|  | Sample | | | | | |
| --- | --- | --- | --- | --- | --- | --- |
|  | A | B | C | D | E | F |
| Parameter |  |  |  |  |  |  |
| Number of  laboratories | 9 | 12 | 12 | 12 | 9 | 9 |
| Number of laboratories after elimination of  outliers | 8 | 11 | 12 | 12 | 8 | 9 |
| Outliers (Cochran  test) | 1 | 1 | - | - | 1 | - |
| Outliers (Grubbs  test) | - | - | - | - | - | - |
| Mean | 0.561 | 2.432 | 1.074 | 1.391 | 1.949 | 0.5327 |
| Reproducibility  standard deviation  (sR) | 0.348 | 0.558 | 0.396 | 0.504 | 0.497 | 0.444 |
| Repeatability  standard deviation  (sr) | 0.325 | 0.077 | 0.266 | 0.246 | 0.180 | 0.216 |
| Relative sR (%) | 62.1 | 22.9 | 36.8 | 36.2 | 25.5 | 83.4 |
| Relative sr (%) | 57.9 | 3.2 | 24.8 | 17.7 | 9.3 | 40.6 |
| Reproducibility,  R (2.80 * sR) | 0.974 | 1.562 | 1.107 | 1.411 | 1.392 | 1.244 |
| Repeatability,  r (2.80 * sr) | 0.909 | 0.217 | 0.745 | 0.689 | 0.505 | 0.605 |

Table S7: Precision data for the differences (Δ δ^13^C (‰), absolute values) between δ^13^C (‰) of disaccharides and δ^13^C (‰) of trisaccharides in honey determined by LC–IRMS.

|  | Sample | | | | | |
| --- | --- | --- | --- | --- | --- | --- |
|  | A | B | C | D | E | F |
| Parameter |  |  |  |  |  |  |
| Number of  laboratories | 9 | 12 | 12 | 12 | 9 | 9 |
| Number of laboratories after elimination of  outliers | 9 | 11 | 11 | 12 | 9 | 9 |
| Outliers (Cochran  test) | - | - | 1 | - | - | - |
| Outliers (Grubbs  test) | - | 1 | - | - | - | - |
| Mean | 0.883 | 0.509 | 0.472 | 0.361 | 2.285 | 1.369 |
| Reproducibility  standard deviation  (sR) | 0.566 | 0.401 | 0.254 | 0.265 | 0.524 | 0.538 |
| Repeatability  standard deviation  (sr) | 0.499 | 0.153 | 0.114 | 0.135 | 0.241 | 0.397 |
| Relative sR (%) | 64.1 | 78.7 | 53.8 | 73.3 | 22.9 | 39.3 |
| Relative sr (%) | 56.5 | 30.0 | 24.1 | 37.4 | 10.5 | 29.0 |
| Reproducibility,  R (2.80 * sR) | 1.584 | 1.122 | 0.711 | 0.741 | 1.467 | 1.507 |
| Repeatability,  r (2.80 * sr) | 1.398 | 0.428 | 0.318 | 0.378 | 0.674 | 1.112 |
